# Tau pathology in early Alzheimer's disease is linked to selective disruptions in neurophysiological network dynamics

**DOI:** 10.1016/j.neurobiolaging.2020.03.009

**Published:** 2020-08

**Authors:** Ece Kocagoncu, Andrew Quinn, Azadeh Firouzian, Elisa Cooper, Andrea Greve, Roger Gunn, Gary Green, Mark W. Woolrich, Richard N. Henson, Simon Lovestone, Deep and Frequent Phenotyping study team, James B. Rowe

**Affiliations:** a Department of Clinical Neurosciences, University of Cambridge, Cambridge, UK; b Oxford Centre for Human Brain Activity, Wellcome Centre for Integrative Neuroimaging, University of Oxford, Oxford, UK; c Department of Psychiatry, University of Oxford, Oxford, UK; d Invicro LLC, London, UK; e MRC Cognition and Brain Sciences Unit, University of Cambridge, Cambridge, UK; f Department of Medicine, Imperial College London, London, UK; g Department of Engineering Science, University of Oxford, Oxford, UK; h Department of Psychology, University of York, York, UK; i Department of Psychiatry, University of Cambridge, Cambridge, UK

**Keywords:** Alzheimer's disease, Connectivity, Graph theory, MEG, Tau, PET

## Abstract

Understanding the role of Tau protein aggregation in the pathogenesis of Alzheimer's disease is critical for the development of new Tau-based therapeutic strategies to slow or prevent dementia. We tested the hypothesis that Tau pathology is associated with functional organization of widespread neurophysiological networks. We used electro-magnetoencephalography with [^18^F]AV-1451 PET scanning to quantify Tau-dependent network changes. Using a graph theoretical approach to brain connectivity, we quantified nodal measures of functional segregation, centrality, and the efficiency of information transfer and tested them against levels of [^18^F]AV-1451. Higher Tau burden in early Alzheimer's disease was associated with a shift away from the optimal small-world organization and a more fragmented network in the beta and gamma bands, whereby parieto-occipital areas were disconnected from the anterior parts of the network. Similarly, higher Tau burden was associated with decreases in both local and global efficiency, especially in the gamma band. The results support the translational development of neurophysiological “signatures” of Alzheimer's disease, to understand disease mechanisms in humans and facilitate experimental medicine studies.

## Highlights

• Alzheimer tau pathology linked to neurophysiological impairments in humans.
• Higher Tau burden is linked to disconnection of posterior parts of the brain.
• Higher Tau burden is linked to declines in local and global efficiency in gamma.
• Eigenvector centrality of the occipital lobe shows declines in only 6 months.

## 1 Introduction

There is a pressing need for new therapeutic strategies to prevent or arrest Alzheimer's disease, especially where applicable at the prodromal stage of disease. The evaluation of new candidate compounds requires robust tools to investigate the pathogenic mechanisms in early stages of disease. Commonly used tools to quantify the effects of Alzheimer's disease pathology in people vary in the degree of invasiveness (e.g., magnetic resonance imaging *versus* lumbar puncture), cost and scalability for large trials (e.g., blood tests *versus* positron emission tomography), and the degree to which they provide mechanistic insight into the pathogenesis of Alzheimer's disease (e.g., cognitive tests *versus* positron emission tomography).

In this study, we take a neurophysiological perspective to link recent advances in preclinical and translational models of Alzheimer's disease. There are 2 key aspects to our approach. First, is the recognition of the effect of Tau and Aβ on synaptic dysfunction (Ittner et al., 2010, Murray et al., 2015), early in the cascade of Alzheimer pathogenesis and before atrophy or cell death. This in turn impairs the network dynamics that underpin cognition (Ahmed et al., 2014, Kimura et al., 2014). Tau and Aβ also induce changes in GABAergic function (Li, G. et al., 2009), and glutamatergic function (Hsieh et al., 2006, LaFerla and Oddo, 2005, Li et al., 2009, Liu et al., 2004, Shankar et al., 2007), further disrupt effective communication in local and large scale neurocognitive networks.

Second, is the recognition of neurophysiological signatures of Alzheimer's disease. For example, magnetoencephalography (MEG) distinguishes Alzheimer's disease pathology from frontotemporal lobar degeneration by their spectral signatures, while retaining functional anatomical concordance with the clinical syndromes (Sami et al., 2018). The brain's evoked and induced responses as measured by MEG and electroencephalography (EEG) distinguish Alzheimer's disease from controls, in advanced disease (Sitnikova et al., 2018), mild cognitive impairment stage (Dauwels et al., 2010, Hughes et al., 2019), and even presymptomatically in carriers of autosomal dominant mutations (Ochoa et al., 2017, Suarez-Revelo et al., 2016). The spectral features of noninvasive clinical studies recapitulate invasive and ex vivo recordings of transgenic model systems (Koss et al., 2016, Kurudenkandy et al., 2014, Sami et al., 2018). Thus, MEG and EEG have potential to capture network dysfunction before extensive brain atrophy. However, the relationship between these physiological indices and Tau pathology in human Alzheimer's disease is unknown.

In the present study, we exploited the spatiotemporal precision of MEG to study network connectivity and oscillatory patterns, across different frequency bands. We use a graph theoretical approach, to extract regional and frequency specific summary measures of complex network function (Bullmore and Sporns, 2009). Graph metrics have been widely investigated in healthy population. In health, the brain displays a fractal-like “small world organization” enabling high global and local efficiency of information transfer (Achard and Bullmore, 2007, Bassett et al., 2006, Smit et al., 2008). Studies show that the healthy function of interconnected modules that make up the larger brain network modules is dependent on age (Fair et al., 2009, Ferreira et al., 2016, Meunier et al., 2009, Meunier et al., 2014, Valencia et al., 2009) and physical fitness (Douw et al., 2014). The brain achieves a balance between segregation and integration (Valencia et al., 2009), in which an area's role in multiple functional modules is measured by its participation coefficient at rest and under cognitive demands (Cohen and D'Esposito, 2016). Furthermore, centrality measures that quantify the functional influence of brain regions in global information transfer are higher in associative “hub” regions (Garcés et al., 2016, Lohmann et al., 2010). The influence of hubs weakens with age (Fan et al., 2017) and neurodegenerative disease (Crossley et al., 2014), causing disruptions in information transfer (Váša et al., 2015).

Alzheimer's pathology severely affects these network connectivity and characteristics. For example, patients show reduced effective connectivity between posterior and anterior regions of the brain in the beta band (Dauwan et al., 2016); higher delta and theta power, and reduced alpha power and peak frequency (Gouw et al., 2017). Similarly asymptomatic amyloid-positive older controls show reduced connectivity within the precuneus and increased connectivity between precuneus and parietal areas in the theta and delta bands (Nakamura et al., 2017). Alzheimer's patients show decreased small worldness (López-Sanz et al., 2017, Vecchio et al., 2016) and decreased local efficiency measured by the clustering coefficient in the alpha and beta bands (de Haan et al., 2009, Stam et al., 2009). Furthermore, the centrality of temporal posterior cortical regions decreases (de Haan et al., 2012b, Yu et al., 2017) and intermodular connectivity weakens (de Haan et al., 2012a) suggesting an imbalance of local functional influence and segregation. Although neurophysiological graph metrics have been intensively used to characterize network abnormalities in Alzheimer's disease, their relationship with Tau pathology remains unclear.

Here we tested the relationship between neurophysiological network properties and the degree of Tau pathology. Previously, MEG-based measures of functional connectivity have been investigated in relation to phosphorylated Tau levels in CSF (Canuet et al., 2015), but CSF Tau measures do not indicate regional variation in Tau pathology across the cortex. Using fMRI based network connectivity measures (rather than MEG/EEG), we have shown that the degree of connectivity of each cortical region correlates with expression of the MAPT gene for Tau (Rittman et al., 2016) and the accumulation of Tau as measured by [^18^F]AV-1451 PET (Bischof et al., 2019, Cope et al., 2018, Hoenig et al., 2018, Sepulcre et al., 2017). This ligand binds to Tau aggregates in Alzheimer's disease, in proportion to disease severity (Brier et al., 2016, Passamonti et al., 2017), and mirrors the distribution of pathology and functional deficits in variant presentations of Alzheimer's disease (Ossenkoppele et al., 2016). We therefore used [^18^F]AV-1451 PET to test the relationship between Tau pathology burden and MEG connectivity.

Our primary goal was to quantify the correlation of Tau burden with neurophysiological network properties in early Alzheimer's disease. A secondary goal was to measure the effect of Tau burden on the rate of change in these network properties, over six months. We focused our analysis on network characteristics that have been widely used to quantify the efficiency of information transfer, internetwork communication and the function of network hubs. We hypothesized that in Alzheimer's disease, (i) efficiency of information transfer at the local and global level is reduced; (ii) the influence of central nodes on the network weakens; (iii) segregation of functional modules is reduced; and (iv) that these changes in graph metrics correlate with local increases in Tau burden across the cortex.

## 2 Materials and methods

### 2.1 Study design

The Deep and Frequent Phenotyping Study is a collaboration between the Dementias Platform UK and the NIHR Translational Research Collaboration in Dementia. It aims to assess the acceptability and feasibility of extensive and frequent phenotyping to aid the design of larger scale future biomarker studies (Koychev et al., 2017). Here, we report data from the pilot study phase, which included patients with early symptomatic Alzheimer's disease. The study was approved by the National Research Ethics Committee London (REC reference 14/LO/1467). All participants had mental capacity and provided written informed consent.

### 2.2 Participants

Twelve patient participants (Table 1) with probable Alzheimer's disease according to National Institute of Aging–Alzheimer's Association criteria (McKhann et al., 2011) were recruited from local memory clinics (mean age: 69.94, age range: 54–82.7, 9 males, 3 females). Patients were amyloid positive based on their CSF Aβ42/40 ratios below 0.09. The NHS Trusts involved in the recruitment were Oxford University Hospitals, South London and Maudsley, Cambridge University Hospitals, University College Hospital London, West London Mental Health Care, and Newcastle Hospitals. Other inclusion criteria were having an Mini-Mental State Examination (MMSE) score of 20 and above, modified Hachinski score of 4 or less, being on stable medication dose for any nonsignificant medical conditions for at least one month, and stable dose for at least 3 months if treated with cholinesterase inhibitors and/or memantine. Participants had a mean MMSE score of 24/30 (*SD =* 2.27), mean CDR score of 0.6/3 (*SD =* 0.31), and mean ADAS-Cog score of 13.9/70 (*SD =* 5.43).

**Table 1:** Sample characteristics

Subject	Gender	Age	MMSE	Global uptake	PET braak
S1	M	54	26	0.26	IV/V
S2	M	69	25	0.22	III/IV
S3	M	74	24	0.13	III/IV
S4	M	61	22	0.26	III/IV
S5	M	77	29	0.19	I/II
S6	F	64	20	0.30	III/IV
S7	F	82	25	0.12	I/II
S8	M	82	24	0.17	III/IV
S9	F	73	22	0.23	IV/V
S10	M	56	24	0.31	III/IV
S11	M	64	25	0.23	III/IV
S12	M	78	23	0.16	III/IV

Key: MMSE, Mini-Mental State Examination; PET, positron emission tomography.

Data from 12 healthy age-matched control participants were taken from an independent data set (mean age: 66.83, age range: 61–75, 8 males, 4 females). Control participants were healthy older adults without any neurological or psychiatric condition, and normal hearing and vision. Healthy controls had a mean MMSE score of 29.25 (*SD =* 0.96). The difference in mean age between the patient and control groups was not significant (*p =* 0.464).

### 2.3 E/MEG acquisition

Scans were acquired at rest (eyes open) over five minutes at 4 sites at the baseline (Functional Imaging Laboratory at University College London, Oxford Centre for Human Brain Activity in University of Oxford, York Neuroimaging Centre at University of York, and MRC Cognition and Brain Sciences Unit at the University of Cambridge) using 3 types MEG scanners (CTF/VSM Omega 275, and Elekta Vector View 306 and 4D Magnes 3600). Simultaneous E/MEG data were acquired in the Cambridge (70-channel Easycap), York (32-channel Neuroscan) and Oxford (64-channel Easycap) sites. For the purposes of the current analysis, we included participants (N = 12) who had complete eyes-open resting state scan, T1-weighted MR scan and [^18^F]AV-1451 scan at the baseline. MEG scans of these participants were acquired in Cambridge, Oxford, and London (Table 1). Nine of these participants had a repeat E/MEG scan 6 months later. Detailed description of the data collected from the patients could be found in Supplementary Information. The E/MEG data of the control participants were acquired in Cambridge, at one time point only for cross-sectional comparison.

Participants were seated in a magnetically shielded room and positioned under the MEG scanner in the upright position. EOG and ECG electrodes were used where available, plus head position indicator coils. For coregistration of the participant's T1-weighted MRI scan to the MEG sensors, 3 fiducial points (nasion, left, and right preauricular) and head surface points were digitized using Polhemus digitization. Simultaneous E/MEG was recorded continuously at 1000 Hz.

### 2.4 PET and MR

PET scans of the patient participants were acquired at Imanova. PET scans were not available for the healthy controls. MR scans of the patients and controls were acquired at the Cambridge, Oxford, and London sites, using Siemens 3T Trio with a 32-channel phased array head coil. 1 mm isotropic whole-brain structural 3D T1-weighted MPRAGE images were acquired using TI = 880 ms, TR = 2000 ms, and FA = 8° with a parallel imaging factor of 2. Two dynamic PET scans for Aβ and Tau were acquired on separate days. Participants were injected an intravenous bolus of [^18^F]AV-1451 (120 minutes, 163 ± 10 MBq) and [^18^F]AV45 tracers (60 minutes, 150 ± 24 MBq) for Tau and Aβ, respectively. A low dose CT scan immediately before each PET scan was used to estimate attenuation. The scans were acquired on Siemens PET/CT scanners (either Hi-Rez Biograph 6 or Biograph 6 TruePoint with TrueV, Siemens Healthcare, Erlangen, Germany). Dynamic images were reconstructed using a 2D filtered back projection algorithm resulting in a 128 × 128 matrix with 2 mm isotropic voxels. Corrections were applied for attenuation, randoms, scatter, and tracer radioactive decay.

**Fig. 1:**
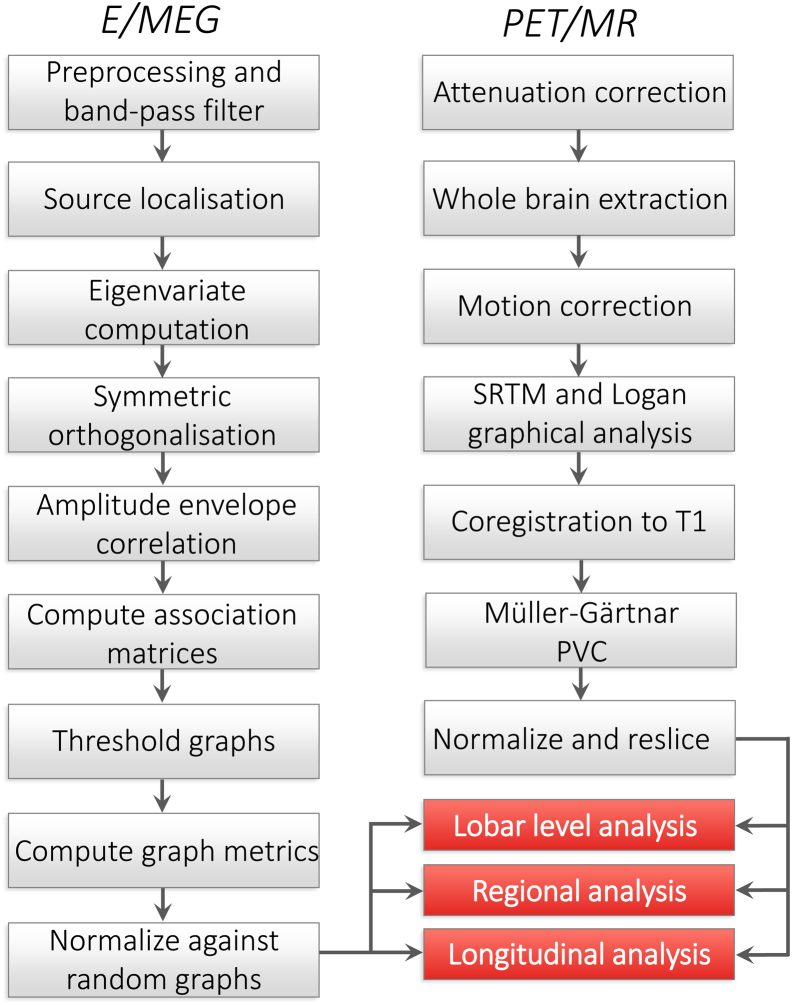
Summary of the analysis: Pipeline showing the steps of E/MEG and PET/MR processing leading up to the graph theoretical analysis. Abbreviations: SRTM, simplified reference tissue model; PVC, partial volume correction.

Summary steps of the PET and MR preprocessing are given in Fig. 1 (Firouzian et al., 2018). PET and MRI imaging processing used MIAKAT (www.miakat.org). Each participant's whole brain was extracted using the FMRIB software library (Jenkinson et al., 2012), brain extraction tool (Smith, 2002), and the corresponding gray matter probability maps were created using SPM5 (www.fil.ion.ucl.ac.uk/spm). Furthermore, dynamic PET data were corrected for motion. Regional time activity curves were generated using the atlas and dynamic PET images. The simplified reference tissue model with cerebellar gray matter as a reference region were applied to the regional time activity curves to estimate the nondisplaceable binding potential (BP_ND_). The resulting BP_ND_ maps were coregistered to participant's T1-weighted MRI scan. To correct for the partial volume effects, the Müller-Gärtner method was applied voxelwise, which uses a 3-compartment model of the brain (i.e., white matter, gray matter and CSF tissue maps), as implemented in the PETPVE12 toolbox (Gonzalez-Escamilla et al., 2017). MR images and corrected PET images were normalized and resliced to match the atlas dimensions and resolution (1 mm isotropic). Current analysis focused on cortical Tau burden only. The [^18^F]AV-1451 uptake values of the patients' medial temporal lobe could be found in Supplementary Information.

T1-weighted MRI scans were further processed using the VBM8 toolbox (http://dbm.neuro.uni-jena.de/vbm8). To calculate local gray matter atrophy, scans were segmented into gray matter, white matter, and CSF tissue maps using the maximum a posterior technique (Rajapakse et al., 1997). Segmentation used a partial volume estimation with a simplified mixed model of at most 2 tissue types for more accurate segmentation (Tohka et al., 2004). To account for intensity inhomogeneity, local variations of the parameters were modeled as slowly varying spatial functions. Gray and white matter segments in native space were then normalized to a DARTEL template using affine registration and the nonlinear DARTEL algorithm (Ashburner, 2007). Finally, gray matter segments were smoothed with a Gaussian kernel at 8 mm full width half maximum.

### 2.5 E/MEG preprocessing and source localization

The raw MEG data acquired through Elekta scanners were preprocessed using MaxFilter 2.2 (Elekta Oy). Maxfiltering included detection and interpolation of bad sensors, signal space separation to remove external noise from the data, and head movement correction. MEG data acquired through the CTF system were analyzed as third-order synthetic gradiometers. Cardiac and blink artifacts were removed using an independent component analysis with 800 maximum steps and 64 principal components via the EEGLAB toolbox (Delorme and Makeig, 2004). On average 1.36 blink components (*SD =* 0.95) and 0.8 cardiac components (*SD =* 0.41) were removed from patients, and 1.9 blink components (SD = 1.2) and 0.6 cardiac components (SD = 0.5) were removed from the controls. Summary steps of the E/MEG preprocessing are given in Fig. 1A.

Data were further processed in SPM12 (www.fil.ion.ucl.ac.uk/spm). Data were bandpass filtered to 5 frequency bands of interest using fifth-order Butterworth filters: delta (0.1–4 Hz), theta (4–8 Hz), alpha (8–12 Hz), beta (12–30 Hz), and gamma (30–100 Hz). Data in the gamma band were further notch filtered to remove line noise. The continuous data were epoched into 4s long consecutive segments, resulting in approximately 75 epochs per participant. These segments were visually inspected for any remaining artifacts (e.g., motor) and bad channels and trials were removed. On average 5.46 (*SD =* 7.39) trials and 2.25 channels (*SD =* 2.75) were removed from patient data and 3.75 (SD = 3.75) trials and 6.93 (SD = 6.75) channels from the control data. Data were then downsampled to 200 Hz.

The E/MEG data were source localized using all sensor types (Henson et al., 2009). The source space was modeled with a medium-sized cortical mesh consisting of 8196 vertices via inverse normalization of SPM's canonical meshes. Sensor positions were coregistered to the native T1-weighted MPRAGE scans using the fiducial and head shape points. Single shell and BEM models were used for forward modeling of MEG and EEG data, respectively. Total power (induced and evoked) was estimated over the trials using the minimum norm estimate solution (R^2^ model fit in patients: *M =* 91.49; *SD =* 6.56; R2 model fit in controls: *M =* 92.49; *SD* = 4.62). Across all participants, the 2 visits and the frequency bands, 5 inversions (of total 165) from the patient's data set showed R^2^ lower than 80%, and were excluded from the following analyses. Among these excluded data, 3 came from the baseline visit delta band, 1 from the baseline theta band, and 1 from 6 month's delta band.

### 2.6 Graph theoretical analysis

A cortical graph was based on the Harvard-Oxford atlas thresholded at 25%, with 98 cortical parcels including the hippocampi. The data were extracted from all the vertices that constitute each parcel, and their first eigenvariate was computed. Multivariate leakage correction method was applied that removes the zero lag effects across all parcels using symmetric orthogonalization (Colclough et al., 2015), allowing a more accurate estimation of functional dependencies. The Hilbert envelope of each parcel's time series was computed to extract the analytic signal, and epochs were concatenated. Pairwise functional connectivity between parcels was computed using amplitude envelope correlations, which was previously shown to be the most consistent network connectivity estimate at the group level, and at the subject-level after leakage correction (Colclough et al., 2016). The amplitude envelope correlations of every pair of parcels formed the association matrices.

Choices of the analysis parameters were made based on test-retest reliability outcomes. The association matrices were thresholded at 25% density. This threshold was chosen because reliability of the metrics at low sparsities (<10%) is low: networks get fractured and disconnected (Dennis et al., 2012). Reliability of the metrics is improved at higher densities (Braun et al., 2012) and have been shown to be stable between sparsities of 0.2–0.3, with a sharp drop in reliability above 0.3 (Dennis et al., 2012). We opted for thresholded weighted graphs as they generate more stable measurements compared with binarized graphs (Wang et al., 2011). Graph metrics were then calculated on the weighted association matrices using the Brain Connectivity Toolbox (Rubinov and Sporns, 2010) in MATLAB 2017a (the Mathworks Inc, 2017). We use 4 metrics to capture essential global and local characteristics of the network communication at the nodal level. Metrics were computed for each of 98 nodes, 5 frequency bands and 12 participants, then normalized against 500 random graphs with equivalent degree.

(1) Eigenvector centrality is an extension of degree centrality. Degree centrality measures how many links connect with a node, giving equivalent weights to links coming from each connecting node. Eigenvector centrality is a meta-metric that quantifies the functional influence of a node on every other node in the graph, by weighting the importance of each nodal connection based on the influence of the nodes with which they connect. It is measured as the first eigenvector of the adjacency matrix corresponding to the largest eigenvalue (Bonacich, 1972).
(2) Clustering coefficient is the fraction of triangular connections formed by a node with other nodes. A node is strongly clustered if a large proportion of its neighbors are neighbors of each other. Because nodes that have high local clustering are also well connected locally, this measure captures local efficiency of information transfer.
(3) Closeness centrality is defined as the inverse of the sum of shortest path lengths between a node and all other nodes in the graph. It is a nodal measure of global efficiency, reflecting long-range efficiency of information transfer and network integration, where low values of closeness centrality indicate low global efficiency of communication.
(4) Participation coefficient reflects the diversity of nodes' intermodular connections (i.e., connectivity to multiple functional modules), and is computed using the Louvain community detection algorithm (Blondel et al., 2008). Participation coefficient captures the segregation of functional networks, where a high participation coefficient would indicate connectivity to a high number of segregated functional modules. Modular networks maintain a balance between functionally specialized modules that have high within and between-module connectivity. Because of this fine balance, higher participation coefficient values do not necessarily correspond to better modularity; they could reflect a breakdown of functional segregation.

### 2.7 ROI selection

To reduce the number of multiple comparisons and to test the relationship between Tau deposition and graph metrics locally, we selected a subset of our 98 nodes. ROIs were selected by taking the areas that show Tau burden above the cortical mean in the group average (M = 0.13; SD = 0.05). These areas were middle frontal gyri, posterior cingulate cortex, hippocampi, cuneal cortex, inferior and superior lateral occipital cortex, occipital, temporal and temporo-occipital fusiform cortices, angular gyri, precuneus, superior parietal lobule, posterior supramarginal gyri, anterior, posterior and temporo-occipital inferior and middle temporal gyri, anterior, and posterior superior temporal gyri bilaterally, adding up to 21 ROIs. ROIs overlap with brain regions widely reported to accumulate neurofibrillary tangles and show atrophy in early stages of Alzheimer's disease (Braak et al., 2006, Jack et al., 2018, Johnson et al., 2016, Ossenkoppele et al., 2016). The neurophysiological metrics, Tau burden, and gray matter atrophy were calculated for each ROI, averaging values across the hemispheres.

### 2.8 Correlations and statistical analysis

We tested the relationship between the network properties and Tau burden both at the lobar level and at the level of the above ROIs. Similar to the approach used in Cope et al. (2018), the lobar level correlations informed us whether there was a linear trend across functional areas. By contrast, the ROI level correlations showed local correlations in areas affected early in the disease. By adopting this two-step approach, we quantified Tau-graph relationship both at the lobar and local level.

To assess the relationship between the Tau burden and cognitive scores (i.e., MMSE, CDR, ADAS-Cog), we used partial correlations controlling for participants' age. The nodes were grouped into 5 functional areas (i.e., lobes): frontal, temporal, parietal, occipital, and limbic areas. Each metric was averaged within the functional lobe. Statistical comparisons were performed using general linear models (GLM), adjusting for the differences in age and MEG acquisition site (Cambridge, Oxford, London). Using GLMs, metric changes at the lobar and ROI level were compared between patients and controls to find disease-related changes in network properties. Later, to investigate the effect of Tau burden on the metrics, the local uptake values were tested against metrics from patients' baseline visit additionally controlling for the local gray matter atrophy. Finally, to explore longitudinal changes in network function, we tested for the difference in the metrics from patients' baseline and 6 months visits. The resulting p values were corrected for multiple comparisons of metrics permutation statistics with 500 permutations. Results are reported at the alpha level of 0.01. The graph metrics in the MNI space were back-projected onto the canonical FreeSurfer cortical surface for ease of visualization using the bspmview toolbox (https://github.com/spunt/bspmview/).

## 3 Results

### 3.1 Tau deposition and cognitive measures

We performed one-tailed Pearson's partial correlations between the total Tau burden and the cognitive scores while controlling for the effect of age. In these correlations, total Tau was calculated as the mean [^18^F]AV-1451 BP_ND_ across all parcels. The whole brain mean [^18^F]AV-1451 uptake was not significantly correlated with the MMSE (*r =* −0.44; *p =* 0.091), whereas the relationship with ADAS-COG (*r =* 0.55; *p =* 0.035) and CDR was significant (*r =* 0.76; *p =* 0.003), confirming the direct relationship between disease severity and cortical Tau burden.

### 3.2 Tau deposition and connectivity

**Fig. 2:**
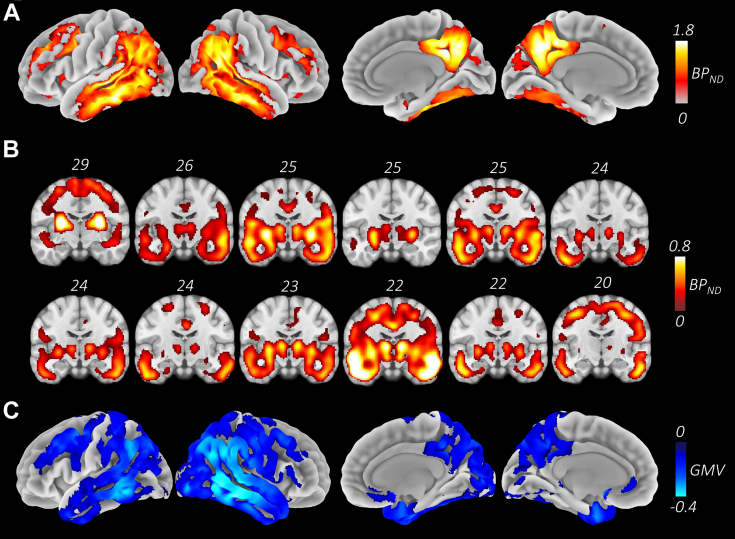
[^18^F]AV-1451 binding and atrophy profiles. A. Map of mean Tau deposition across the patients, measured as the [^18^F]AV-1451 nondisplaceable binding potential where lighter colors indicate higher Tau burden across the sample. The map shows the characteristic Alzheimer's disease distribution that spreads over temporo-parietal, posterior medial and superior frontal areas bilaterally. Strongest overlap is around the precuneus, angular gyri, posterior middle temporal, and inferior temporal areas. B. Tau deposition maps of individual participants ordered by decreasing MMSE scores, displaying the left lateral and medial views. C. Group atrophy map showing the gray matter differences between patient and control groups. (For interpretation of the references to color in this figure legend, the reader is referred to the Web version of this article.)

All patient participants showed the typical widespread bilateral Tau deposition (Passamonti et al., 2017, Schöll et al., 2016). Fig. 2A shows the mean [^18^F]AV-1451 BP_ND_ maps across the patient group, with highest levels at the precuneus, posterior cingulate, posterior middle temporal, anterior fusiform, inferior parietal lobules, and the putamen. Fig. 2B shows the [^18^F]AV-1451 BP_ND_ binding, ordered from left to right in decreasing MMSE scores. In line with the whole brain correlation results, we did not observe an inverse correlation between tau burden and MMSE, although all patients showed tau accumulation in the medial temporal lobe. Fig. 2C shows the mean gray matter atrophy in the patient group.

Before the graph analysis, we tested for changes in undirected functional connectivity at the lobar level. Group comparisons showed significant increased connectivity in the patient group between limbic and occipital (*t* (15) = 2.55; *p =* 0.008) and limbic and parietal lobes (*t* (15) = 2.57; *p =* 0.008) in the delta band only. Increase in delta band connectivity suggests oscillatory slowing in the patient group.

### 3.3 Group differences between the healthy controls and patients

Differences in graph metrics were tested between patients and controls at each lobe and ROI across the frequency bands. At the lobar level, we found significant decreases in patients in closeness centrality in parietal beta (*t* (18) = −2.89; *p =* 0.004), and significant decreases in eigenvector centrality in frontal gamma (*t* (18) = −2.48; *p =* 0.006) and parietal beta (*t* (18) = −3.38; *p =* 0.002). The eigenvector centrality decrease in the gamma band was significant also at the whole brain level (*t* (18) = −2.31; *p =* 0.008). There were further decreases at the uncorrected level in clustering and participation coefficient in beta and gamma bands.

The results at the ROI level mirrored the lobar level results. We found a significant decrease of closeness centrality in the patient group in posterior supramarginal gyrus beta (*t* (18) = −3.33; *p =* 0.002). There were widespread decreases in eigenvector centrality in the beta band in middle frontal gyrus (*t* (18) = −2.97; *p =* 0.002), cuneus (*t* (18) = −2.80; *p =* 0.008), inferior lateral occipital cortex (*t* (18) = −3.42; *p =* 0.006), superior parietal lobule (*t* (18) = −3.60; *p =* 0.002), and angular gyrus (*t* (18) = −4.07; *p =* 0.002). In gamma band, there were significant decreases in the anterior inferior temporal gyrus (*t* (18) = −2.63; *p =* 0.004). In addition, we found a significant increase in the patient group in anterior middle temporal gyrus delta (*t* (12) = 3.22; *p =* 0.004). Clustering coefficient was significantly lower in the patient group in superior parietal lobule (*t* (18) = −2.95; *p =* 0.008) and angular gyrus beta (*t* (18) = −2.81; *p =* 0.004). Participation coefficient showed decreases in the gamma band across the ROIs only at the uncorrected level. Overall, the group comparisons indicate reduced efficiency of information transfer both at local and global levels in parietal areas in the beta band. Whereas functional influence of a node shows widespread reductions across the brain in beta and gamma bands.

### 3.4 Effect of Tau burden on network properties

**Fig. 3:**
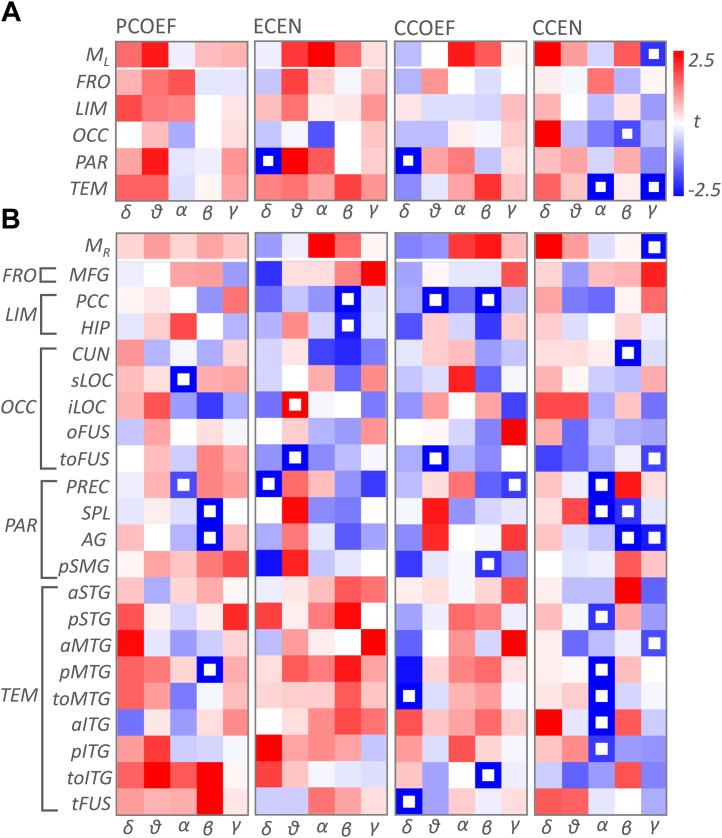
T-maps showing the relationship between Tau levels and the graph metrics. A. T-maps at the lobar level where rows and columns indicate lobes and frequency bands, respectively. Top row, M_L_, shows the t values for the whole brain. White boxes show significant relationship at *p* = 0.01 level (corrected). B. T-maps at the ROIs that have high Tau burden. Top row, M_R_, shows the t values for the mean of the ROIs. Abbreviations: PCOEF, participation coefficient; ECEN, eigenvector centrality; CCOEF, clustering coefficient; CCEN, closeness centrality; FRO, frontal; LIM, limbic; OCC, occipital; PAR, parietal; TEM, temporal; γ, gamma; β, beta; α, alpha; θ, theta; δ, delta band; MFG, middle frontal gyrus; PCC, posterior cingulate cortex; HIP, hippocampus; CUN, cuneus; sLOC, superior lateral occipital cortex; iLOC, inferior lateral occipital cortex; oFUS, occipital fusiform; toFUS, temporo-occipital fusiform; PREC, precuneus; SPL, superior parietal lobe; AG, angular gyrus; pSMG, posterior supramarginal gyrus; aSTG, anterior superior temporal gyrus; pSTG, posterior superior temporal gyrus; aMTG, anterior middle temporal gyrus; pMTG, posterior middle temporal gyrus; toMTG, temporo-occipital middle temporal gyrus; aITG, anterior inferior temporal gyrus; pITG, posterior inferior temporal gyrus; toITG, temporo-occipital inferior temporal gyrus; tFUS, temporal fusiform.

We tested the relationship between the graph metrics and local Tau deposition in the patient group at the lobar and the ROI level using GLMs. Results of these tests are given in Fig. 3. At the lobar level, we found a negative relationship between Tau and closeness centrality in temporal alpha (*t* (6) = −3.96; *p =* 0.002) and gamma (*t* (6) = −2.44; *p =* 0.002). The negative relationship in gamma was also significant at the whole brain level (*t* (6) = −1.97; *p =* 0.008). Parietal delta also showed a negative relationship with Tau for the clustering coefficient (*t* (3) = −5.71; *p =* 0.006) and the eigenvector centrality (*t* (3) = −4.74; *p =* 0.006).

**Fig. 4:**
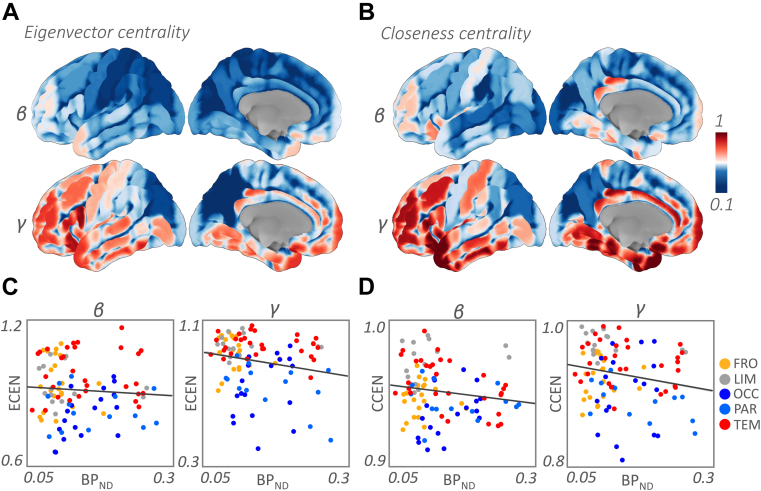
Tau-related change in eigenvector and closeness centrality. (A–B). Cortical rendering of eigenvector and closeness centrality in beta and gamma bands, respectively. (C–D). Scatterplots showing the relationship between the metrics and Tau uptake for the group mean of the metrics and Tau levels for all the nodes. Plots show that the centrality values are lower on the posterior part of the brain, in the inferior parietal lobule and occipital cortex both medially and laterally. Abbreviations: ECEN, eigenvector centrality; CCEN, closeness centrality; FRO, frontal; LIM, limbic; OCC, occipital; PAR, parietal; TEM, temporal; γ, gamma; β, beta; BP_ND_, nondisplaceable binding potential.

At the ROI level, we found negative relationship between the closeness centrality and Tau widespread across alpha, beta, and gamma bands. In the alpha band, the effects were observed in the precuneus (*t* (6) = −3.61; *p =* 0.004), superior parietal lobule (*t* (6) = −5.47; *p =* 0.002), posterior superior temporal gyrus (*t* (*6*) = −2.03; *p =* 0.002), posterior middle temporal gyrus (*t* (6) = −6.16; *p =* 0.002), temporo-occipital middle temporal gyrus (*t* (6) = −3.43; *p =* 0.002), anterior (*t* (6) = −2.81; *p =* 0.004), and posterior inferior temporal gyrus (*t* (6) = −1.91; *p =* 0.008). In the beta band, we found a negative relationship in the cuneus (*t* (6) = −5.30; *p =* 0.004), superior parietal lobule (*t* (6) = −1.96; *p =* 0.004), and angular gyrus (*t* (6) = −2.89; *p =* 0.002). In the gamma band, the negative effect was significant across the ROIs (*t* (6) = −2.34; *p =* 0.002), and in temporo-occipital fusiform cortex (*t* (6) = −1.79; *p =* 0.006), angular gyrus (*t* (6) = −2.21; *p =* 0.008), and anterior middle temporal gyrus (*t* (6) = −1.77; *p =* 0.006). Similarly, eigenvector centrality showed negative relationship with Tau in the theta band temporo-occipital fusiform cortex (*t* (5) = −9.52; *p =* 0.002), and in the beta band, posterior cingulate cortex (*t* (6) = −2.86; *p =* 0.008) and hippocampus (*t* (6) = −2.24; *p =* 0.006). Fig. 4 shows the cortical renderings of the eigenvector and closeness centrality in the beta and gamma bands, and their relation to the Tau burden.

Furthermore, we found effects for the clustering coefficient in temporo-occipital middle temporal gyrus (*t* (3) = −2.61; *p =* 0.009) and temporal fusiform cortex (*t* (3) = −2.60; *p =* 0.006) in the delta band; posterior cingulate cortex (*t* (5) = −2.72; *p =* 0.004) and temporo-occipital fusiform cortex in the theta band (*t* (5) = −4.34; *p =* 0.002); posterior cingulate cortex (*t* (5) = −2.75; *p =* 0.004) and temporo-occipital inferior temporal gyrus (*t* (6) = −2.67; *p =* 0.001) in the beta band and precuneus in the gamma band (*t* (6) = −1.93; *p =* 0.009). The effects observed for the participation coefficient were restricted to the alpha and beta band. In the alpha band, we found significant negative relationship with Tau in the superior lateral occipital cortex (*t* (6) = −3.28; *p =* 0.002) and precuneus (*t* (6) = −1.73; *p =* 0.009). In the beta band, the effects were in superior parietal lobule (*t* (6) = −2.74; *p =* 0.006), angular gyrus (*t* (6) = −4.56; *p =* 0.002), and posterior middle temporal gyrus (*t* (6) = −2.58; *p =* 0.004).

### 3.5 Longitudinal changes in network properties and connectivity

The connectivity changes within the lobes displayed only subtle changes in magnitude and were not significant at the corrected level. We then compared the change in lobar means across 6 months in the patient group. At the lobar level, we found significant decrease in eigenvector centrality in the occipital lobe (*t* (10) = −2.90; *p =* 0.008), and increased closeness centrality in the frontal lobe (*t* (10) = 2.57; *p =* 0.008) in the delta band.

ROI analyses showed further decreases in the eigenvector centrality in the delta band in inferior lateral occipital cortex (*t* (10) = −4.06; *p =* 0.004), precuneus (*t* (10) = −3.65; *p =* 0.002), and posterior middle temporal gyrus (*t* (10) = −3.77; *p =* 0.002). Closeness centrality was decreased in the precuneus delta band (*t* (10) = −2.34; *p =* 0.008). Similarly, we found decreased clustering coefficient in the temporo-occipital fusiform cortex in the theta band (*t* (10) = −2.85; *p =* 0.004) and in posterior superior temporal gyrus in the beta band (*t* (12) = −3.49; *p =* 0.002). Finally, participation coefficient increased in the temporo-occipital fusiform cortex (*t* (10) = 2.68; *p =* 0.008) in the theta band, hippocampus (*t* (12) = 2.86; *p =* 0.002) in the alpha band, and posterior supramarginal gyrus (*t* (12) = 3.01; *p =* 0.004) and posterior superior temporal gyrus in the beta band (*t* (12) = 3.38; *p =* 0.002).

## 4 Discussion

The aim of the present study was to investigate changes in neurophysiological network properties in early Alzheimer's disease in relation to regional Tau burden. We calculated graph metrics that capture nodal levels of global and local efficiency in information transfer, functional influence of a node on the remaining network (i.e., “hubness”), and functional segregation of the modules. We found widespread Tau-related decreases in eigenvector centrality in occipital and parietal areas, suggesting and anterior-posterior breakdown of communication and a shift toward a more fragmented network topology. Second, Alzheimer's patients showed significant declines in both local and global efficiency of information transfer with increasing Tau burden. Declines in functional influence and global-level communication affected higher frequency bands, whereas functional connectivity in the lower frequency bands increased, showing an oscillatory slowing. Finally, the comparisons of metrics across just 6 months showed further decreases in centrality and efficiency, the most prominent being the decrease in occipital eigenvector centrality in the delta band. Our findings indicate the potential for neurophysiological markers in experimental medicines studies or early phase trials in Alzheimer's disease.

E/MEG has been widely utilized in brain network research, moving out of its historical use in epilepsy to elucidate the neurophysiological underpinnings of cognition and dysfunction (da Silva, 2013). Despite its lower spatial resolution compared with magnetic resonance imaging, E/MEG has an advantage in that it can directly measure neuronal activity with millisecond time resolution and without convolution by vascular signaling (Tsvetanov et al., 2015, Tsvetanov et al., 2016). This fast capture allows one to see transient patterns of brain activity that are invisible to other methods (Singh, 2012). Moreover, it allows investigations of brain activity patterns, spread across different frequency bands (Başar et al., 2001). The current analyses demonstrate this multifaceted nature of neurophysiology, revealing how Alzheimer's disease affects information embedded in different frequencies.

We found widespread decreases in both eigenvector and closeness centrality in the patient group. Eigenvector centrality measures the functional, “hub-like”, influence of regions exerted on the remaining network. We found that compared with the controls, patients declines particularly in the parietal and occipital areas, in the cuneus, lateral occipital cortex, superior parietal lobule, and angular gyrus. This pattern has been recently reported using fMRI, related to the CSF p-Tau level as well as patients' MMSE scores (Binnewijzend et al., 2014, Cope et al., 2018). Similarly, the glucose metabolism and eigenvector centrality of the occipital and parietal areas of the ApoE4 carriers gets reduced (Adriaanse et al., 2016, Luo et al., 2017, Ossenkoppele et al., 2013) and synchronization likelihood of the occipital areas decrease (Sanz-Arigita et al., 2010). Furthermore, ApoE4 carriers show reduced posterior default mode connectivity in cognitive normal aging adults (Machulda et al., 2011). In patients, reduced connectivity between the anterior and posterior components of the default mode network, and reduced connectivity within the posterior default mode areas was reported (Liu et al., 2014). A meta-analysis of fMRI studies show hypoactivation in the occipital cortex in Alzheimer's patients (Li et al., 2015). The occipital and parietal Tau-related reductions in eigenvector centrality, suggest a diminished role of posterior part of the brain in Alzheimer's disease, and a shift toward an increasingly fragmented network where parietal and occipital nodes get further disconnected and isolated. Studies report an inverse pattern in frontal areas suggesting a shift of balance in the network. Such that frontal areas show higher eigenvector centrality among Alzheimer's patients and ApoE4 carriers (Binnewijzend et al., 2014, Luo et al., 2017) and their synchronization likelihood increases (Sanz-Arigita et al., 2010). However, this opposing fronto-occipital pattern was not observed in our study after correcting for site-specific changes.

In the healthy brain network, the connectivity between the brain regions are in an economical balance such areas have high local and global efficiency, with “small-world” topology. We report Tau-related decreases in global efficiency, as measured by closeness centrality, in the occipital, parietal, and more prominently in the temporal areas. These findings are in line with prior studies that show increased characteristic path length (i.e., decreased global efficiency) in functional (Stam et al., 2007) and white matter connectivity (Fischer et al., 2015, Tuladhar et al., 2016). Furthermore, lower global efficiency at the baseline was associated with increased risk of dementia after 5 years (Tuladhar et al., 2016).

Similarly, we found reductions in local efficiency, measured by the clustering coefficient, in the superior parietal lobule and angular gyrus in patients compared with the controls. The local efficiency negatively correlated with Tau burden in parietal and temporal areas. Prior studies report mixed results on clustering. Some studies show lower clustering in Alzheimer's disease both functionally and structurally (Supekar et al., 2008, Zhou and Lui, 2013), whereas others show preserved (Stam et al., 2007), or increased clustering coefficient (Cope et al., 2018). These mixed results could be due to the differences in disease stages of the patient populations across studies. Overall, these reductions of both global and local efficiency indicate a shift away from the small world topology to an ordered topology. The healthy cognitive function in older adults depends on maintaining connectivity within and between large-scale networks (Tsvetanov et al., 2016), increasing the fault tolerance of the network to disease (Strogatz, 2001). The move away from a protective connectivity profile in early Alzheimer's disease might therefore accelerate disease progression, with further network breakdown and cognitive decline.

One of the advantages of MEG is to the ability to investigate disease-related changes across frequency bands, in a way that cannot be done with MRI. We found reductions in eigenvector and closeness centrality in the beta and gamma bands. Differences in eigenvector centrality were in the frontal, occipital, and parietal areas. While closeness centrality effects in gamma were at the whole brain level. Long-range gamma synchrony has been proposed to be fundamental to integrate information processed in-tandem across regions in a network (Başar-Eroglu et al., 1996), such that reductions in functional connectivity in the higher frequency bands reflect loss of small world topology (de Haan et al., 2012b). Synchronization of processing in the gamma band occurs both locally and across long distances in the cortex even with zero lag delays (Rodriguez et al., 1999, Singer, 1999). Previous studies reported widespread loss of long-range gamma synchrony in humans (Koenig et al., 2005, Stam et al., 2002) and tau-mediated network instability in gamma band in mouse models of Alzheimer's disease (Verret et al., 2012). We speculate that a contributor to the changes in the gamma band is degeneration of cholinergic projections which enhance these frequencies in the healthy brain (Rodriguez et al., 2004).

At lower frequency ranges, in the delta band, we found increased connectivity between limbic, occipital, and parietal areas, which in healthy adults strongly oscillate in the alpha, beta (Mantini et al., 2007), and theta bands (de Pasquale et al., 2010). Together with the Tau-related network changes observed in higher frequency bands, this indicates that in early Alzheimer's disease internetwork communication shifts from higher to lower frequency bands. These results are complementary to reported increases in delta and theta synchrony (Babiloni et al., 2004, Poza et al., 2008), where the slowing of frequencies was related to the white matter atrophy (Babiloni et al., 2006) and progression from mild cognitive impairment to Alzheimer's disease (Babiloni et al., 2010, Huang et al., 2000, Jelic et al., 2000). Compared with Alzheimer's patients who are either ϵ2 or ϵ3 carriers, ϵ4 carriers display longitudinal increases in delta and theta power (Lehtovirta et al., 1996). This slowing could be linked to the impairments in cholinergic-muscarinic transmission which causes decreases in gamma, and increases in resting delta and theta power (Bosboom et al., 2009).

Compared with controls, Alzheimer's disease was associated with reduced participation coefficient in the gamma band. Tau-related decreases in participation coefficient were observed in the alpha and beta bands in posterior temporal and parietal areas. These results are in line with the findings showing decreasing participation coefficient, modularity, and intermodular connectivity in the frontal, occipital, and parietal regions in mild cognitive impairment and Alzheimer's disease (Brier et al., 2014, de Haan et al., 2012a). Cope et al. further reported a decrease in participation coefficient with increasing Tau (Cope et al., 2018), where occipital and parietal nodes displayed the strongest Tau-related drop in participation. The decrease in participation indicates a decline of multimodule connectivity and increasing regional isolation with more severe Tau pathology. However, cross-sectional correlations with severity cannot be interpreted as evidence of within-subject progression.

The second aim of the study was to quantify changes that occur over 6 months in patients. Changes in such a short period could aid the faster assessment of experimental drugs for Alzheimer's disease. In the delta band, we found further interval decreases in eigenvector centrality in the occipital lobe and increased closeness centrality in the frontal lobe, indicating further disconnection between anterior and posterior parts of the brain. Increased global efficiency in the frontal lobe could be attributed to a potential compensatory mechanism in reduced global efficiency of the parietal and occipital areas. The ROI level comparisons revealed further decreases in clustering coefficient, and in contrast to Tau-graph analyses, increases in participation coefficient in temporo-parietal areas. This analysis also highlighted key regions that show faster rates of change in their network properties over 6 months. We found that the network properties changed faster in the precuneus, temporo-occipital fusiform cortex, posterior middle and superior temporal gyri, and lateral occipital cortex. This could be attributed to their faster rates of Tau accumulation (Ishiki et al., 2015) and of cortical thinning observed for the mild cognitive impairment patients converting to Alzheimer's disease and Alzheimer's patients (Li et al., 2012).

A limitation of the study was the small sample size and the absence of longitudinal PET data. To overcome these limitations, we adopted an exploratory and correlational approach to the Tau burden and neurophysiological metrics. However, future studies with bigger sample sizes are needed to confirm the findings of the present study. The inclusion of patients at different stages of Alzheimer's disease, even as a larger group, would not support inferences about individual longitudinal progression. Longitudinal studies, including longitudinal PET, would enable a formal mediation analysis of the pathology and physiological and functional progression of Alzheimer's disease. However, larger studies would enable one to test nonlinear progression of pathophysiology. The inclusion of other measures of pathology, such as PET assays of synaptic density, may add sensitivity and insights to early-stage neurophysiological consequences of Alzheimer's disease. However, the E/MEG is arguably more scalable across sites for cost-effective and safely repeated assessment of disease and the effect of experimental medicine.

## 5 Conclusions

Our findings provide preliminary evidence that Tau pathology is associated with human brain network connectivity in specific spectral bands. This may arise from synaptic dysfunction as shown in animal models of Alzheimer's disease, neurotransmitter deficits, or additionally from somatic and axonal deficits. Higher Tau burden in early Alzheimer's disease is associated with a more fragmented network where parieto-occipital areas are disconnected from the remaining network and a shift away from the optimal small-world organization. Furthermore, Alzheimer's disease changed markedly the long-range global efficiency of temporal cortex. The neurophysiological network biomarkers that relate to Tau pathology may be especially useful as noninvasive tools to track short-term disease progression and the impact of disease modifying therapies on brain function.

## Disclosure statement

The authors declare that they have no competing interests.

## CRediT authorship contribution statement

**Ece Kocagoncu:** Methodology, Formal analysis, Data curation, Visualization, Writing - original draft. **Andrew Quinn:** Investigation. **Azadeh Firouzian:** Investigation, Formal analysis. **Elisa Cooper:** Investigation. **Andrea Greve:** Investigation. **Roger Gunn:** Conceptualization, Methodology. **Gary Green:** Resources. **Mark W. Woolrich:** Methodology, Supervision, Writing - review & editing. **Richard N. Henson:** Methodology, Supervision, Writing - review & editing. **Simon Lovestone:** Conceptualization, Funding acquisition, Writing - review & editing. **James B. Rowe:** Methodology, Conceptualization.
