# Supplementary material for: Tau pathology in early Alzheimer's disease is linked to selective disruptions in neurophysiological network dynamics
Source: Neurobiol Aging. 2020 Aug;92:141–52. doi: 10.1016/j.neurobiolaging.2020.03.009 (PMC7269692; doi:10.1016/j.neurobiolaging.2020.03.009)
Supplement: Supplement [file mmc1.docx]

# Supplemental Information

***Table S1. Data description***

| ***Subject*** | ***Site*** | ***Scanner*** | ***Baseline MEG*** | ***Baseline PET/MR*** | ***6 Months MEG*** |
| --- | --- | --- | --- | --- | --- |
| **S1** | **Cambridge** | **Elekta Neuromag** | **Yes** | **Yes** | **Yes** |
| **S2** | **Cambridge** | **Elekta Neuromag** | **Yes** | **Yes** | **Yes** |
| **S3** | **Cambridge** | **Elekta Neuromag** | **Yes** | **Yes** | **Yes** |
| **S4** | **Cambridge** | **Elekta Neuromag** | **Yes** | **Yes** | **Yes** |
| **S5** | **London** | **CTF** | **Yes** | **Yes** | **Yes** |
| **S6** | **London** | **CTF** | **Yes** | **Yes** | **No** |
| **S7** | **London** | **CTF** | **Yes** | **Yes** | **Yes** |
| **S8** | **London** | **CTF** | **Yes** | **Yes** | **Yes** |
| **S9** | **Oxford** | **Elekta Neuromag** | **Yes** | **Yes** | **No** |
| **S10** | **Oxford** | **Elekta Neuromag** | **Yes** | **Yes** | **Yes** |
| **S11** | **Oxford** | **Elekta Neuromag** | **Yes** | **Yes** | **No** |
| **S12** | **Oxford** | **Elekta Neuromag** | **Yes** | **Yes** | **Yes** |

***Table S2. Levels of AV-1451 BP_ND_ across the sample***

| ***Subject*** | ***Global tau uptake*** | ***Uptake in hippocampus*** | ***Uptake in entorhinal cx*** |
| --- | --- | --- | --- |
| **S1** | **0.26** | **0.21** | **0.14** |
| **S2** | **0.22** | **0.19** | **0.10** |
| **S3** | **0.13** | **0.13** | **0.16** |
| **S4** | **0.26** | **0.16** | **0.09** |
| **S5** | **0.19** | **0.09** | **0.04** |
| **S6** | **0.30** | **0.12** | **0.07** |
| **S7** | **0.12** | **0.10** | **0.08** |
| **S8** | **0.17** | **0.09** | **0.11** |
| **S9** | **0.23** | **0.12** | **0.13** |
| **S10** | **0.31** | **0.11** | **0.13** |
| **S11** | **0.23** | **0.15** | **0.14** |
| **S12** | **0.16** | **0.17** | **0.12** |
| **Mean** | **0.21 ± 0.06** | **0.14 ± 0.04** | **0.11 ± 0.03** |
